# Did you see the sound? A Bayesian assessment on cross-modal perception in low vision

**DOI:** 10.1167/jov.26.8.7

**Published:** 2026-08-25

**Authors:** Ailene Y. C. Chan, Noelle R. B. Stiles, Carmel A. Levitan, Armand R. Tanguay, Shinsuke Shimojo

**Affiliations:** 1 California Institute of Technology, Division of Biology and Biological Engineering, Pasadena, CA, USA; 2 Rutgers University, Departments of Neurology, Ophthalmology and Visual Science, Biomedical Engineering; Center for Advanced Human Brain Imaging Research in the Brain Health Institute, New Brunswick, NJ, USA; 3 Occidental College, Cognitive Science, Los Angeles, CA, USA; 4 University of Southern California, Departments of Electrical Engineering, Chemical Engineering and Materials Science, Biomedical Engineering, Ophthalmology, and Physics and Astronomy; Neuroscience Graduate Program, Los Angeles, CA, USA

**Keywords:** vision, low vision, audition, multisensory, illusion, Bayesian causal inference

## Abstract

Multisensory integration is often assumed to increase when visual input is degraded, yet it remains unclear whether low vision enhances susceptibility to cross-modal illusions or whether such effects depend on local variations in visual reliability. We tested low-vision and sighted control participants on the sound-induced flash illusion (SIFI) across 24 visual-field locations, concurrently measuring baseline flash-detection accuracy. While both groups showed the expected auditory-driven increase in perceived flash numerosity, sighted controls were significantly more susceptible to the classic “double-flash” percept. While illusion strength approached marginal significance for eccentricity, it was more robustly predicted by local flash-detection accuracy, indicating that sound-induced percepts depend on the availability of a reliable visual signal. Exploratory Bayesian causal inference modeling revealed that when fitted across all locations, model performance was systematically weaker and more variable for low-vision observers. Crucially, however, restricting the analysis to high-visibility locations yielded robust model fits by the Bayesian information criterion and close agreement between observed and predicted response proportions. This suggests that, like sighted controls, low-vision participants continue to behave in a manner broadly consistent with Bayesian-optimal inference when visual information is sufficiently reliable. Together, these findings demonstrate that low vision does not globally amplify audiovisual interactions; rather, multisensory enhancements are constrained by local visual reliability, and Bayesian-like inference remains most evident when provided with a sufficient threshold of sensory input.

## Introduction

In every waking moment, the nervous system must translate incomplete and noisy sensory input into a coherent percept. When multiple senses are simultaneously perceived, the brain must also infer whether multisensory information originates from a single cause or from separate events. A healthy nervous system accomplishes this by weighing sensory cues according to their reliability and by incorporating prior beliefs (or heuristics) about how sensory events unfold (Ernst & Banks, 2002; Körding et al., 2007). In this framework, perception reflects the statistically optimal combination of cues given their uncertainties and the observer's expectations.

But how do these computational principles adapt to chronic sensory degradation? While multisensory integration is well characterized in healthy observers, much less is known about how these mechanisms operate when a primary sense is pathologically compromised. Previous research using experimental manipulations of visual contrast in healthy sighted participants has shown that while the visual likelihood (σ_*V*_) adjusts to accommodate reduced reliability, internal priors (e.g., the prior for common causality, *p*_*common*_) remain remarkably stable (Beierholm, Quartz, & Shams, 2009). This stability is perhaps expected in healthy observers whose daily experience is dominated by high-fidelity visual input.

However, it remains unclear whether these principles hold when visual degradation is not an experimental manipulation, but rather a permanent, pathological state. Low vision offers a particularly informative case: Rather than a complete loss of vision, patients retain islands of intact visual function interspersed with regions of reduced sensitivity. This raises an important question: Does the visually impaired brain globally reorganize its multisensory strategy, or does it continue to adjust integration locally based on the immediate reliability of the visual signal at specific retinal locations?

Neural evidence suggests that the visually impaired brain undergoes cross-modal reorganization, with auditory and tactile signals recruiting occipital cortex even when some visual function remains (Cunningham, Weiland, Bao, Lopez-Jaime, & Tjan, 2015; Norman & Thaler, 2019). Anatomical studies further reveal direct projections from auditory cortex to early visual regions (Beer, Plank, & Greenlee, 2011; Falchier, Clavagnier, Barone, & Kennedy, 2002; Quinones et al., 2022), especially representing peripheral visual space. These findings raise the possibility that cross-modal influences on perception may be enhanced in low vision. However, behavioral findings regarding this enhancement remain mixed. Some evidence suggests that sound can facilitate visual detection in the low-vision group (Targher, Occelli, & Zampini, 2012), yet other studies have found that individuals with severe visual impairment perform worse on tasks requiring complex multisensory integration (Occelli, Spence, & Zampini, 2013). It remains a mystery whether such possible enhancement (or reduction) depends on global differences in sensory processing (e.g., reduced weighting of vision) or local differences driven by the visibility of specific retinal regions.

To address this gap, we used the sound-induced flash illusion (SIFI) (Shams, Kamitani, & Shimojo, 2000), a classic demonstration in which auditory beeps alter the perceived number of visual flashes. We presented the illusion at 24 locations across the visual field in both low-vision and sighted control participants. Our study aimed to determine (a) if low-vision participants exhibit altered susceptibility to the illusion compared to controls, (b) how multisensory interactions vary across the visual field, and (c) whether illusion strength is tied to local visual sensitivity.

While the Bayesian causal inference (BCI) framework has been successfully applied to healthy multisensory perception (Aller & Noppeney, 2019; Rohe & Noppeney, 2015; Shams & Beierholm, 2022), its applicability to chronic clinical populations remains less explored. We therefore adopted an exploratory computational approach, applying BCI modeling to different spatial partitions of the visual field. This allowed us to formally test whether the increased illusion susceptibility often attributed to low vision is a product of altered prior expectations (*p*_*common*_) or a direct consequence of increased sensory uncertainty (σ_*V*_) within a preserved Bayesian architecture.

## Methods

### Human participants

We first recruited 21 neurotypical participants (11 females; mean age = 26.3 years, range = 18–40 years) from the California Institute of Technology community. All had normal or corrected-to-normal vision (20/20) and hearing. Recruitment took place during the early phase of the study, when COVID-19 restrictions limited access to external participants.

We later recruited 25 individuals with low vision after obtaining written informed consent, either through an e-reader or verbally through the experimenter. Four participants did not complete the full experimental protocol and were excluded from data analysis. The final low-vision cohort included 21 participants (8 females; mean age = 51.5 years, range = 25–81 years), all with normal or corrected-to-normal hearing. Demographic and clinical details are shown in Table 1. To account for variations in visual-field sensitivity across participants, particularly given the lack of recent clinical perimetry for low vision individuals, we implemented a custom visual flash detection task. This task evaluated detection accuracy at each of the 24 stimulus locations used in the SIFI task. These data (provided in Supplementary Data Set 1) served as a functional baseline to verify visibility at each stimulus location as well as the eccentricity levels tested (5°, 10°, and 15°).

**Table 1.** Demographic and clinical information of low-vision cohort.

Participant	Diagnosis	Age (years)	Sex	Age of onset (years)	Duration (years)
LV001	Retinitis pigmentosa	65	F	31	34
LV002	Microphthalmia	43	M	0	43
LV003	NAION, right eye	74	M	68	6
LV005	Congenital cataracts	69	M	0	43
LV007	Macular degeneration	70	F	68	2
LV008	Choroideremia	33	M	23	10
LV009	Stargardt’s macular degeneration	28	M	14	14
LV011	Left phthisis bulbi, right cystoid macular edema, uveitis	66	F	24	42
LV012	Glaucoma, right eye total blind	56	M	42	14
LV013	Glaucoma, right eye total blind	40	M	35	5
LV015	Retinitis pigmentosa, detached retina, right eye only	57	M	11	46
LV016	Chronic relapsing inflammatory optic neuritis (CRION)	52	F	36	16
LV017	Enlarged optic nerves	33	M	27	6
LV018	Dominant optic atrophy	25	F	12	13
LV019	Glaucoma, left eye only	81	M	76	5
LV020	Retinal degeneration	42	M	0	42
LV021	Retinitis pigmentosa	41	F	21	20
LV022	Nonarteritic anterior ischemic optic neuropathy (NAION)	64	M	56	8
LV023	Undeveloped optic nerve	57	F	0	57
LV024	Optic nerve damage due to trauma	42	F	3	39
LV025	Microphthalmia	44	M	0	44

After testing the low-vision group, we recruited an additional cohort of age-matched neurotypical controls to better match the age distribution of the low-vision participants. Five participants from the original control cohort were retained because their ages already fell within the target range of the low-vision group and therefore met the age-matching criteria. To complete the age-matched sample, 16 new control participants were recruited, resulting in a final control cohort of 21 participants (15 females; mean age = 51.0 years, range = 24–80 years).

Participants were compensated at $25/hour. All procedures were approved by the institutional review board at the California Institute of Technology (IRB IR20-1025). All experiments were performed at the California Institute of Technology.

### Stimuli and conditions

#### Visual flash-detection task

**Figure 1.**
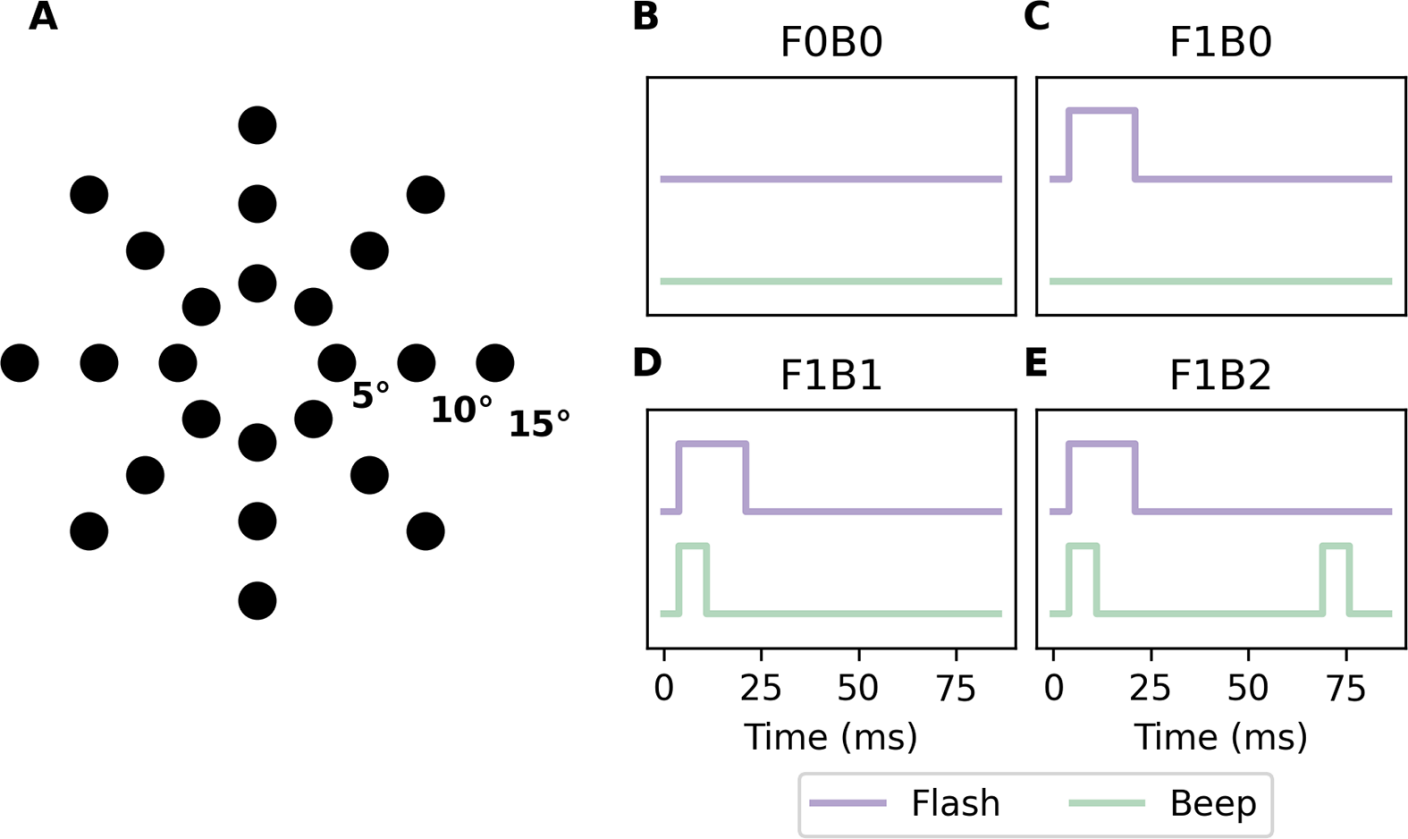
Stimuli and conditions. **(A)** Stimulus locations corresponding to eight equally spaced positions along the circumference at 5°, 10°, and 15° eccentricities from central fixation. **(B–E)** Schematic of the four main experimental conditions, named using an FXBY convention (*X* = number of flashes, *Y* = number of beeps). When paired, visual flashes (16.7 ms) and auditory beeps (7 ms) had simultaneous onsets.

Visual stimuli were circular white discs (2° in diameter) presented on a black background at 100% contrast for one frame (16.7 ms at 60 Hz). Stimuli appeared at 24 possible locations, corresponding to eight equally spaced positions along the circumference at 5°, 10°, and 15° eccentricities from central fixation (Figure 1A). Two audiovisual conditions were tested in this task, following the naming convention FXBY, where X denotes the number of flashes and Y denotes the number of beeps such as F0B0 (no flashes or beeps) and F1B0 (one flash, no beeps). The stimulus timing for these conditions is illustrated in Figures 1B and 1C.

#### SIFI task

This task used the same visual stimuli and stimulus locations as described above (Figure 1A). Auditory stimuli were pure tones (800 Hz, 7-ms duration). Two audiovisual conditions were tested: F1B1 and F1B2. The F1B2 condition, also known as the SIFI condition, elicits the perception of a second illusory flash induced by the second auditory beep. Timing of the stimuli is shown in Figures 1D and 1E.

### Experimental design

#### Visual flash-detection task

The task was performed monocularly for each eye in randomized order, with the nontested eye covered by an eyepatch. Participants indicated whether they detected a flash (*Yes*: press left arrow button; *No*: press right arrow button). The F1B0 condition was presented five times per stimulus location, and the F0B0 condition served as a single catch trial. All trials were randomized.

#### SIFI task

The task was also performed separately for each eye with a randomized order, with the nontested eye covered by an eyepatch. Participants reported the number of perceived flashes (0–3). The prompt “How many flash(es)?” was designed to be linguistically neutral, explicitly allowing for both singular and plural percepts without biasing the participant toward either. The F1B2 condition was presented 10 times per stimulus location, and the F1B1 condition served as a single catch trial. All trials were randomized.

### Experimental setup

Stimuli were presented using Psychtoolbox 3.0.17 in MATLAB 2021a. Auditory tones (62 dB SPL) were delivered via stereo speakers (Bose Companion 2 Series III) placed beside the monitor. Visual stimuli appeared on a 27-inch LCD display (Dell UltraSharp U2720Q; 60 Hz). Participants sat 50 cm from the display with their head stabilized on a chin rest and the nontested eye occluded. The experiment took place in a darkened room. All experimental scripts are publicly available under a CC-BY license (Chan, 2025).

### Data analysis

#### Average perceived flash count by task and stimulus location

**Figure 2.**
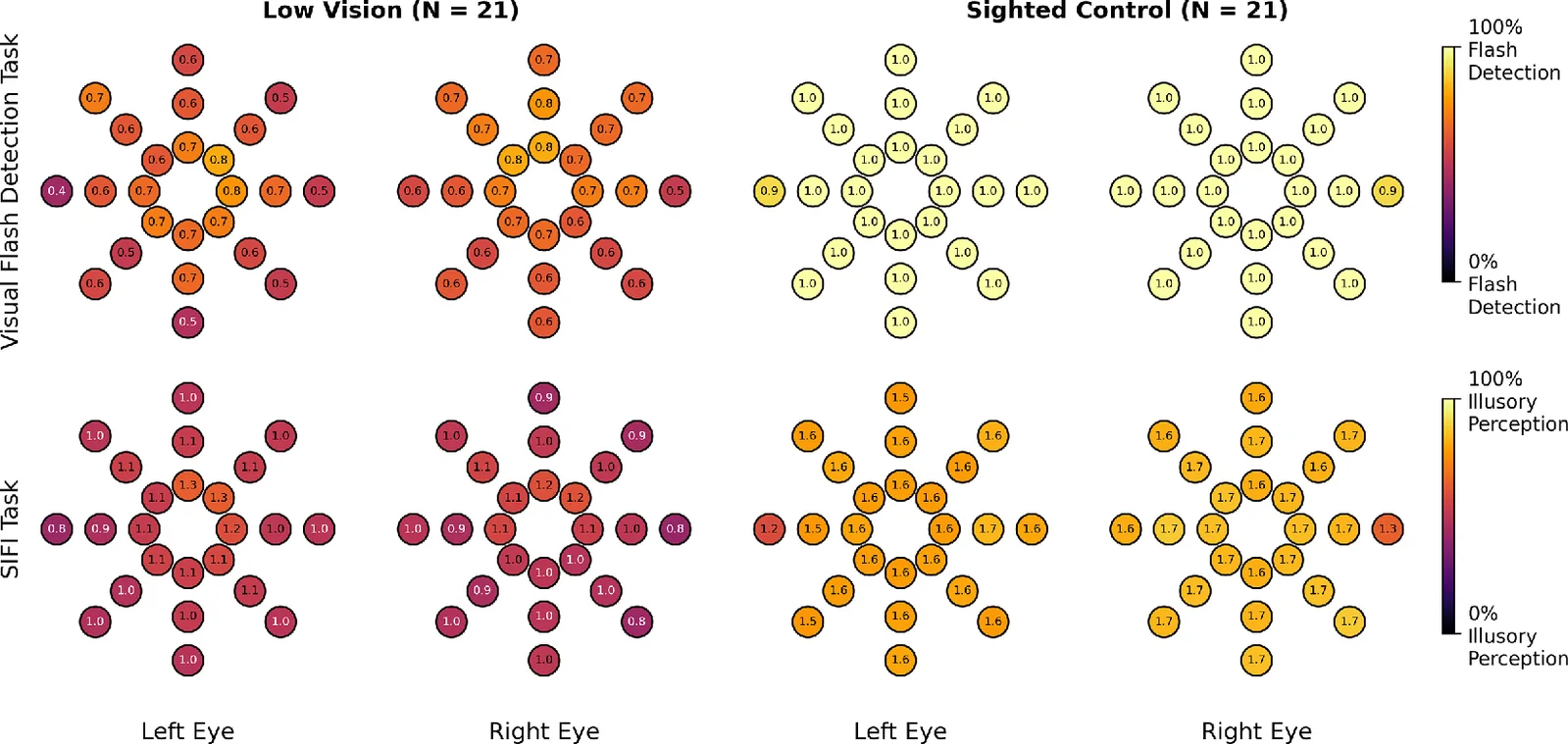
Mean number of flashes perceived by task across all stimulus locations. Top row: *Visual flash-detection task* (F1B0 condition). Bottom row: *Illusory double-flash task* (F1B2 condition). Left panel: Low-vision group. Right panel: Sighted control group. Stimulus locations were further categorized for each participant as high-visibility (flash-detection accuracy >50%) or low-visibility (⩽50%) based on performance in the detection task.

To visualize overall perceptual patterns, we first computed the mean number of flashes reported at each stimulus location for both the visual flash detection (F1B0 trials) and SIFI (F1B2 trials) tasks (Figure 2). Locations were further categorized individually for each participant as high-visibility (flash-detection accuracy >50%) or low-visibility (⩽50%) based on performance in the detection task (i.e., correctly reporting the presence and absence of a flash in F1B0 unimodal trials). To establish the baseline visual accuracy and the presence of the illusion, two Wilcoxon signed-rank tests were conducted within each group to compare perceived flash counts between the baseline (F1B0) and the illusion (F1B2) conditions, with data pooled across all stimulus locations and both eyes. To control for multiple comparisons, Bonferroni correction was applied across the two tests.

#### Accuracy

**Figure 3.**
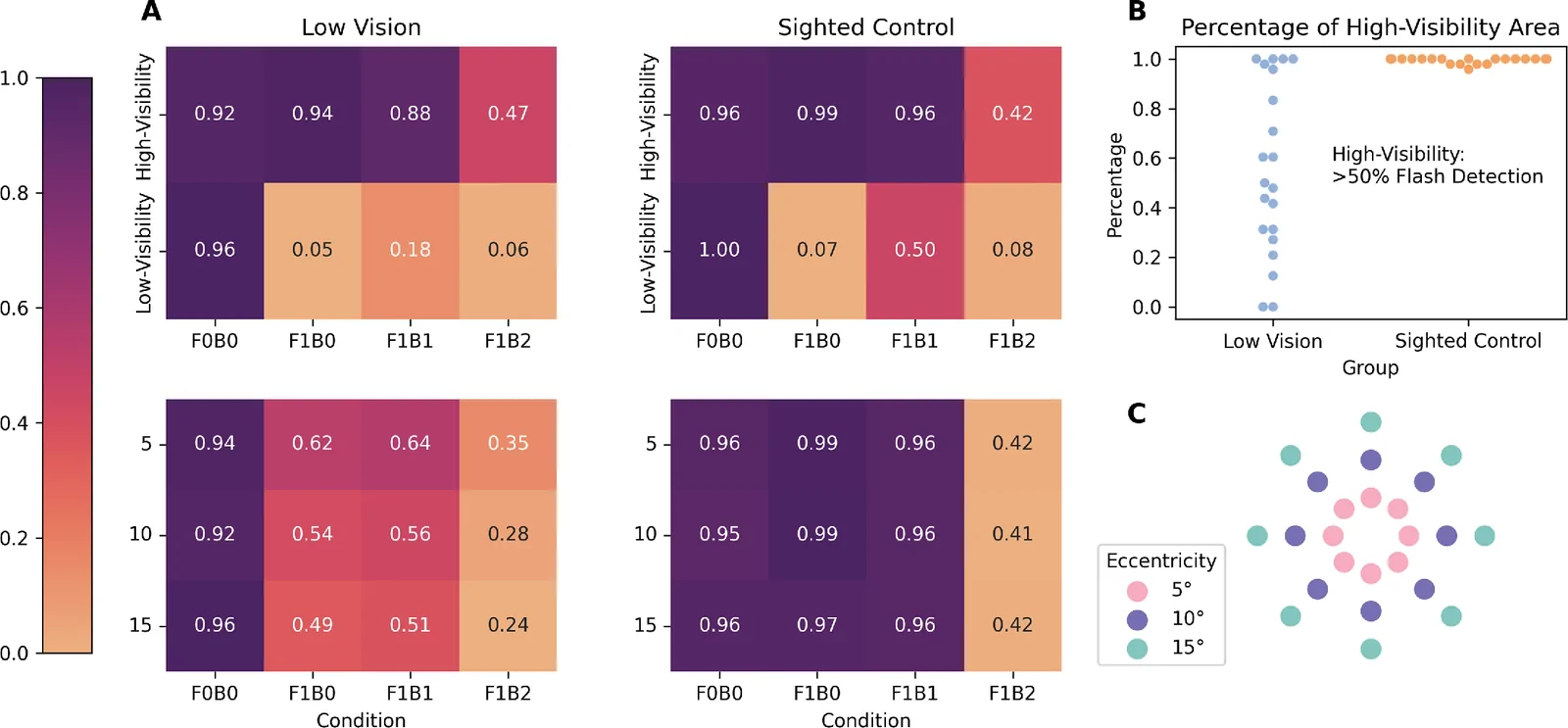
Mean accuracy of flash perception across conditions. **(A)** Heatmaps illustrate the mean accuracy in reporting the veridical number of flashes (0 or 1) across experimental conditions. Locations were categorized into high-visibility (flash-detection accuracy >50%) and low-visibility (⩽50%) cohorts based on individual performance in the visual flash detection task (top panels). Bottom panels display accuracy as a function of stimulus eccentricity (5°, 10°, and 15°). Notably, in the F1B2 (1 flash, 2 beeps) condition, higher accuracy reflects a reduction in the sound-induced flash illusion. Inaccuracy among low-vision participants was split between “misses” (0-flash) and “illusions” (2-flashes), whereas inaccuracy among sighted control participants was driven by a shift toward reporting “illusions” (2-flashes). F0B0 trials yielded the highest overall accuracy, reflecting reliable reports of zero flashes in the absence of stimulation. (**B**) Proportion of stimulus locations classified as high-visibility for each participant. (**C**) Spatial layout of stimulus locations, color-coded by eccentricity (5° = pink, 10° = purple, 15° = green), provided for illustrative purposes.

Accuracy was defined as the proportion of trials in which participants correctly reported the true number of flashes presented (Figure 3A). To examine how visual and spatial factors influenced visual accuracy, we fit two separate linear mixed-effects models using the *statsmodels* package in Python. In both models, the dependent variable was mean flash perception accuracy, and we included a random intercept for participants to account for individual differences in overall accuracy. The first model tested the effects of *group* (low vision, sighted control), *condition* (F0B0, F1B0, F1B1, F1B2), and *visibility* (high-visibility, low-visibility), along with all interactions among these factors. The second model tested the effects of *group*, *condition*, and spatial *eccentricity* (5°, 10°, 15°) and their interactions.

#### Flash detection vs. perceived flash count (F1B1, F1B2)

We examined whether sounds influenced perceived flash numerosity correlates with visual sensitivity (i.e., flash-detection accuracy in the F1B0 condition). For each participant, eye, and stimulus location, we first computed flash-detection accuracy in the F1B0 condition, yielding 48 data points per participant (24 locations × 2 eyes). We then computed, separately for each beep numerosity (B1, B2), the mean number of flashes perceived at each location and eye, again yielding 48 data points per beep condition. To assess the relation between visual sensitivity and sound-driven changes in perceived flash numerosity, we correlated flash-detection accuracy with the mean number of flashes reported using Pearson's correlation coefficient, computed separately for each group (low-vision, sighted control) and beep condition (B1, B2).

#### Model fitting

Participants' responses were modeled using a BCI framework (Körding et al., 2007), from which the forced-fusion, full-segregation, and maximum-likelihood estimation (MLE) models (Ernst & Banks, 2002) were derived as special cases. Model fitting was implemented via the BCI Toolbox (Zhu, Beierholm, & Shams, 2024a) using its API for automated batch processing. To ensure parameter convergence and resolve discrepancies between the distributed package and the source code, we implemented several custom bug fixes; our modified version of the toolbox and all associated analysis scripts are publicly available under a CC-BY licensee (Chan, 2025).

In the BCI model, each sensory event (*s*_*i*_) gives rise to a noisy observation (*x*_*i*_), sampled from a Gaussian centered on the true stimulus value with modality-specific uncertainty (σ_*V*_, σ_*A*_; Equation 1). Observers also hold a prior expectation about stimulus numerosity, modeled as a Gaussian with mean μ_*P*_ and standard deviation σ_*P*_ (Equation 2).

(1) $$x_{V}∼N\left(s_{V},\sigma_{V}\right),\;x_{A}∼N\left(s_{A},\sigma_{A}\right)$$

(2) $$x_{P}∼N\left(\mu_{P},\sigma_{P}\right)$$

Because these Gaussian distributions are continuous, we mapped the model’s internal estimates to the discrete nature of the experimental task. Following the BCI toolbox implementation, internal estimates were rounded to the nearest integer and clipped to the range of possible response values [0, 3].

The model infers whether visual and auditory signals arise from a common (*C* = 1) or independent (*C* = 2) cause by combining sensory likelihoods with a causal prior (*p*_common_) via Bayes' rule (Equation 3). When signals are inferred to share a common cause, the perceived numerosity is a reliability-weighted average of the visual, auditory, and prior estimates; if inferred as independent, each modality is estimated separately.

(3) $$\;\begin{array}{c} p(C=1|x_{V},x_{A})\\ \;=\frac{p\left(x_{V},x_{A}|C\;=\;1\right)p_{\text{common}}}{p\left(x_{V},x_{A}|C\;=\;1\right)p_{\text{common}}+p\left(x_{V},x_{A}|C\;=\;2\right)\left(1-p_{\text{common}}\right)} \end{array}$$

Observers combine these estimates across causal structures (*C* = 1, 2) using one of three decision strategies: *model averaging* (weighted by posterior probability), *model selection* (choosing the more probable cause), or *probability matching* (stochastic selection in proportion to posterior probability) (Wozny, Beierholm, & Shams, 2010).

##### Parameter constraints and validation

To maintain model parsimony, several parameters were fixed based on empirical evidence. The auditory likelihood (σ_*A*_) was set to 0.2, consistent with recent work using identical stimulus configurations in spatiotemporal variants of the SIFI (Chan et al., 2025; Stiles, Li, Levitan, Kamitani, & Shimojo, 2018). To validate this choice for our specific population, we measured beep-detection thresholds in a subset of age-matched controls (*N* = 16). The resulting mean fitted likelihood (σ_*A*_ = 0.19 ± 0.08) was highly consistent with our fixed value (see Appendix S1). Because only flash responses were collected from the main experimental group, auditory responses were simulated by drawing from a Gaussian distribution centered at the true stimulus count (Equation 1) using the fixed σ_*A*_.

To investigate the spatial stability of these processes, we fitted each of the four models (BCI, forced-fusion, full-segregation, and MLE) across six distinct stimulus location subsets for each participant: (a) all locations combined, (b) high-visibility locations, (c) low-visibility locations, and (d) eccentricities at 5°, 10°, and 15°. This hierarchical approach allowed us to evaluate model performance as a function of both sensory reliability and eccentricity. Free and fixed parameters for each model variant are summarized in Table 2.

**Table 2.** Overview of models and their parameters.

Model	Free parameters	#	Fixed parameters
Bayesian causal inference	*p*_common_, σ_*P*_, μ_*P*_, σ_*V*_	4	σ_*A*_
Forced-fusion	σ_*P*_, μ_*P*_, σ_*V*_	3	σ_*A*_, *p*_common_ = 1
Full-segregation	σ_*P*_, μ_*P*_, σ_*V*_	3	σ_*A*_, *p*_common_ = 0
Maximum-likelihood estimation	σ_*V*_	1	σ_*A*_, *p*_common_ = 1, σ_*P*_, μ_*P*_

*Note*: Fixed parameters were set to σ_*A*_ = 0.2 for all models. For MLE, μ_*P*_ = 1.5 and σ_*P*_ = 4,000.

#### Model evaluation

Model performance was evaluated using the Bayesian information criterion (BIC) for model selection and the coefficient of determination (*R*^2^) as a descriptive index of agreement between observed and predicted response proportions. BIC was prioritized because it accounts for varying model complexity ($BIC=k\ln\left(n\right)-2\ln\left(\hat{L}\right)$). In contrast, *R*^2^ was computed over the pooled response-proportion cells across stimulus conditions and response categories:

(4) $$R^{2}=1-\frac{\sum {\left(y_{obs}-y_{pred}\right)}^{2}}{\sum {\left(y_{obs}-{\bar{y}}_{obs}\right)}^{2}}$$

where *y*_*obs*_ and *y*_*pred*_ represent the observed and predicted response proportions across all experimental conditions, respectively. Because this calculation was applied to condition-level response proportions rather than trial-level responses, it should not be interpreted as the proportion of trial-level behavioral variance explained by the model. In the present design, many cells correspond to highly constrained control responses (e.g., near-zero reports of three flashes, near-zero flash reports when no flash was presented, and predominantly one-flash reports for F1B0 trials). These cells contribute substantially to the denominator of *R*^2^ and can inflate the resulting value relative to the theoretically critical illusion cell, namely, the frequency of two-flash reports in the F1B2 condition. We therefore interpret *R*^2^ only as a summary measure of response-proportion agreement and place primary emphasis on BIC and visual comparisons between observed and predicted response distributions.

The robustness of the fixed auditory parameter was verified through a parameter sweep (σ_*A*_ ∈ {0.1, 0.2, 0.3, 0.4, 0.6, 0.8}; see Supplementary Figure S1). Group-level fits peaked within the 0.1 to 0.3 range, with a sharp decline in goodness-of-fit for values ⩾0.4. Statistical comparisons (Wilcoxon signed-rank tests, Bonferroni-corrected) confirmed that ${\text{Model}}_{\sigma_{A}=0.3}$ performed significantly worse than ${\text{Model}}_{\sigma_{A}=0.2}$ across both BIC and *R*^2^ metrics (*p*_*adj*_ < 0.001). While ${\text{Model}}_{\sigma_{A}=0.1}$ yielded lower BIC scores than ${\text{Model}}_{\sigma_{A}=0.2}$ (*p*_*adj*_ < 0.001) for the control group, it offered no significant improvement in *R*^2^ (*p*_*adj*_ = 0.26). For the low-vision group, ${\text{Model}}_{\sigma_{A}=0.1}$ offered no improvement (both BIC and *R*^2^ *p*_*adj*_ = 1). Given this lack of increased predictive power and the alignment of ${\text{Model}}_{\sigma_{A}=0.2}$ with our control group's empirical thresholds, we retained ${\text{Model}}_{\sigma_{A}=0.2}$ for all primary analyses.

Finally, we assessed whether stimulus visibility or spatial location constrained model interpretability. To allow for direct comparison across datasets of varying sizes, normalized BIC scores (*BIC*/*n*) were calculated for every model variant at each spatial location group. For each participant and location subset, this was defined as the raw BIC score divided by the total number of trials (*n*) included in that specific fit (e.g., *BIC*/300 for a 300-trial subset).

## Results

### Average perceived flash count

Participants perceived more flashes during the illusory double flash (F1B2) task (low vision: 1.03 ± 0.15; sighted control: 1.61 ± 0.1) than during the visual flash detection (F1B0) task (low vision: 0.55 ± 0.07; sighted control: 0.98 ± 0.01), consistent with an auditory-induced enhancement of visual perception (Figure 2). This effect was significant in both groups (both Bonferroni-corrected *p* < 0.001). However, only sighted control participants exhibited the full SIFI, reporting an average of 1.61 ± 0.1 flashes across all stimulus locations. Low-vision participants reported an average of 1.03 ± 0.15 flashes.

Together, these findings indicate that while auditory cues increased perceived flash counts in both groups, the canonical SIFI—perceiving two flashes in response to one flash paired with two beeps—was reliably experienced only by sighted controls.

### Accuracy

We defined accuracy as the proportion of trials in which participants correctly reported the exact number of *flashes* presented (0 or 1; Figure 3A). For F1BY conditions (one physical flash present), it is important to note that accuracy reflects a combination of “missed” responses (0 flashes perceived), “correct” responses (1 flash perceived), and illusory responses (2 or 3 flashes perceived). To examine how auditory context, spatial location, and group membership influenced these measures, we fit two linear mixed-effects models with *participant identity* included as a random effect. Both models used *accuracy* as the dependent variable. Fixed effects included *group* (low vision, sighted control), *condition* (F0B0, F1B0, F1B1, F1B2), and *stimulus location*.

The spatial fixed effects were structured as follows:

• **Model 1:** Locations were binned by *functional visibility* (high-visibility vs. low-visibility), defined by each participant's baseline performance (Figure 3B).
• **Model 2:** Locations were binned by *eccentricity* (5°, 10°, 15°) to assess the effect of retinal position regardless of individual visibility thresholds (Figure 3C).

Accuracy differed systematically across auditory conditions and visibility levels (Figure 3B). The mixed-effects model revealed a significant main effect of condition (*p* < 0.001), with participants performing most accurately in the no-flash baseline (F0B0) and showing progressively lower accuracy as the number of accompanying beeps increased (F1B0 > F1B1 > F1B2). High-visibility locations yielded slightly higher accuracy than low-visibility ones, though this main effect of visibility did not reach significance (*p* = 0.28), likely due to ceiling-level performance in the F0B0 baseline across all locations. However, visibility strongly interacted with condition (all *p* < 0.001): Accuracy was significantly higher for high-visibility than low-visibility locations when one flash is physically present (F1B0: β = 0.91, *p* < 0.001; F1B1: β = 0.66, *p* < 0.001; F1B2: β = 0.42, *p* < 0.001). No significant main effects or interactions involving group were observed, indicating that low-vision and sighted control participants showed similar patterns of performance.

We next examined how flash-detection accuracy varied as a function of spatial eccentricity (Figure 3C). The mixed-effects model revealed a significant main effect of condition (*p* < 0.05): Accuracy was highest in the no-flash baseline (F0B0) and declined as the number of beeps increased (F1B2: β = −0.520, *p* < 0.001; F1B1: β = −0.220, *p* = 0.018; F1B0: β = −0.242, *p* = 0.01). Although the main effect of eccentricity was not significant (*p* = 0.78), several two-way interactions between condition and eccentricity hovered near the threshold for significance (F1B0: β = −0.015, *p* = 0.089; F1B1: β = −0.015, *p* = 0.092; F1B2: β = −0.013, *p* = 0.14). These negative coefficients (β ≈ −0.015) suggest a trend toward decreased accuracy in the periphery under specific multisensory constraints. While these comparisons were likely underpowered to reach standard significance levels at our current sample size (*N* = 42), they suggest that eccentricity's impact may be mediated by the reliability of the auditory context. Notably, all three-way interactions involving group, condition, and eccentricity were clearly nonsignificant (*p* > 0.26), suggesting that the impact of eccentricity did not differ between the sighted control and low-vision populations. A modest interaction between group and condition emerged for the F1B0 condition (β = 0.30, *p* = 0.024) and a marginal trend for the F1B1 condition (β = 0.23, *p* = 0.079), indicating slightly higher accuracy for sighted controls in these specific auditory contexts. Beep-detection accuracy was near ceiling across all audiovisual conditions. Because this task was performed only by a subset of sighted control participants, it was not included in the main analyses. The corresponding heatmap is presented in Appendix S1.

To further investigate why accuracy in the F1B2 (illusion) condition remained low for both groups despite differing mean flash reports, we examined the trial-level response distributions (Supplementary Figure S2). For sighted controls, inaccuracy in the F1B2 condition was primarily driven by overreporting (reporting 2 flashes), the hallmark of the SIFI. In contrast, for the low-vision group, inaccuracy was driven by a high frequency of under reporting (reporting 0 flashes).

As shown in the F1B1 and F1B0 control conditions, low-vision participants frequently failed to detect the flash entirely at certain stimulus locations (≈0.5–0.6 accuracy when locations were binned by eccentricity). Consequently, in the F1B2 condition, their mean perceived flashes (reported previously as ∼1.03) does not represent high accuracy but rather an average of frequent “misses” (0 flashes) and occasional “illusions” (2 flashes). These distributional data clarify that while both groups show reduced accuracy in the F1B2 condition, the underlying cause for the low-vision group is a visual detection floor rather than a heightened susceptibility to the auditory-induced illusion.

Together, these results demonstrate that while both groups exhibited similar patterns of overall accuracy, the underlying drivers of performance were distinct. For sighted controls, errors were primarily illusory, reflecting robust cross-modal integration. In contrast, performance in the low-vision group was fundamentally constrained by a visual detection floor, where frequent “misses” (0 flashes) masked any potential gains in resistance to the illusion. Ultimately, flash numerosity accuracy in this task is determined by an interaction between auditory context and localized stimulus visibility, rather than a global shift in multisensory strategies across the visual field.

### Flash detection vs. perceived flash count (F1B1, F1B2)

**Figure 4.**
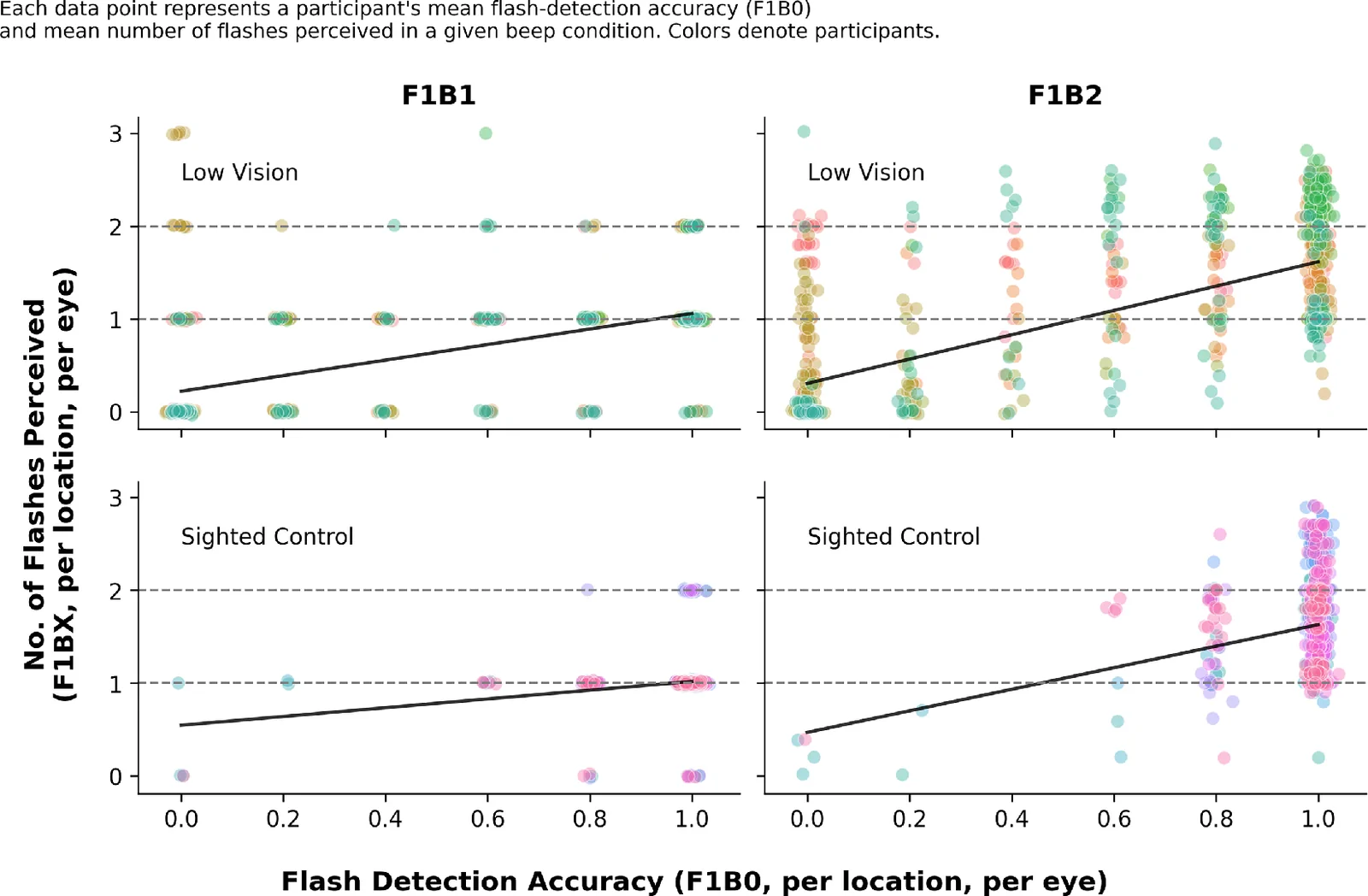
Correlation between flash-detection accuracy and perceived flash counts. Scatterplots show the relationship between flash-detection accuracy (F1B0; visual flash detection task) and the average number of flashes perceived in the one-beep (F1B1, left) and two-beep (F1B2, right) conditions in the illusory double flash task. Each data point represents a participant's detection accuracy and their perceived flash count at a specific stimulus location and eye (48 data points per participant: 24 locations × 2 eyes). In the F1B1 condition (left), data points represent discrete judgments from individual catch trials presented at each location. In the F1B2 condition (right), data points represent the mean perceived flash count across 10 repetitions. Top row: low vision. Bottom row: sighted control.

We next examined whether individual differences in flash-detection accuracy correlated with the strength of sound-induced flash perception. For each participant, eye, and stimulus location, flash-detection accuracy in the F1B0 condition was correlated with the mean number of flashes reported in the one-beep (F1B1) and two-beep (F1B2) conditions (Figure 4).

Both groups showed significant positive correlations between flash-detection accuracy and perceived flash numerosity for both one-beep (low vision: *r* = 0.65, *p* < 0.001; sighted control: *r* = 0.21, *p* < 0.001) and two-beep (low vision: *r* = 0.70, *p* < 0.001; sighted control: *r* = 0.21, *p* < 0.001) conditions. However, visual inspection revealed substantial individual variability, particularly among low-vision participants, suggesting that while higher flash-detection accuracy generally predicted stronger sound-induced flash enhancement, the strength of this relationship varied markedly across observers.

Together, these results indicate that auditory-driven increases in perceived flash numerosity were present in both groups but expressed with considerable individual variability in magnitude.

### Model fitting

#### Model comparison and selection

**Figure 5.**
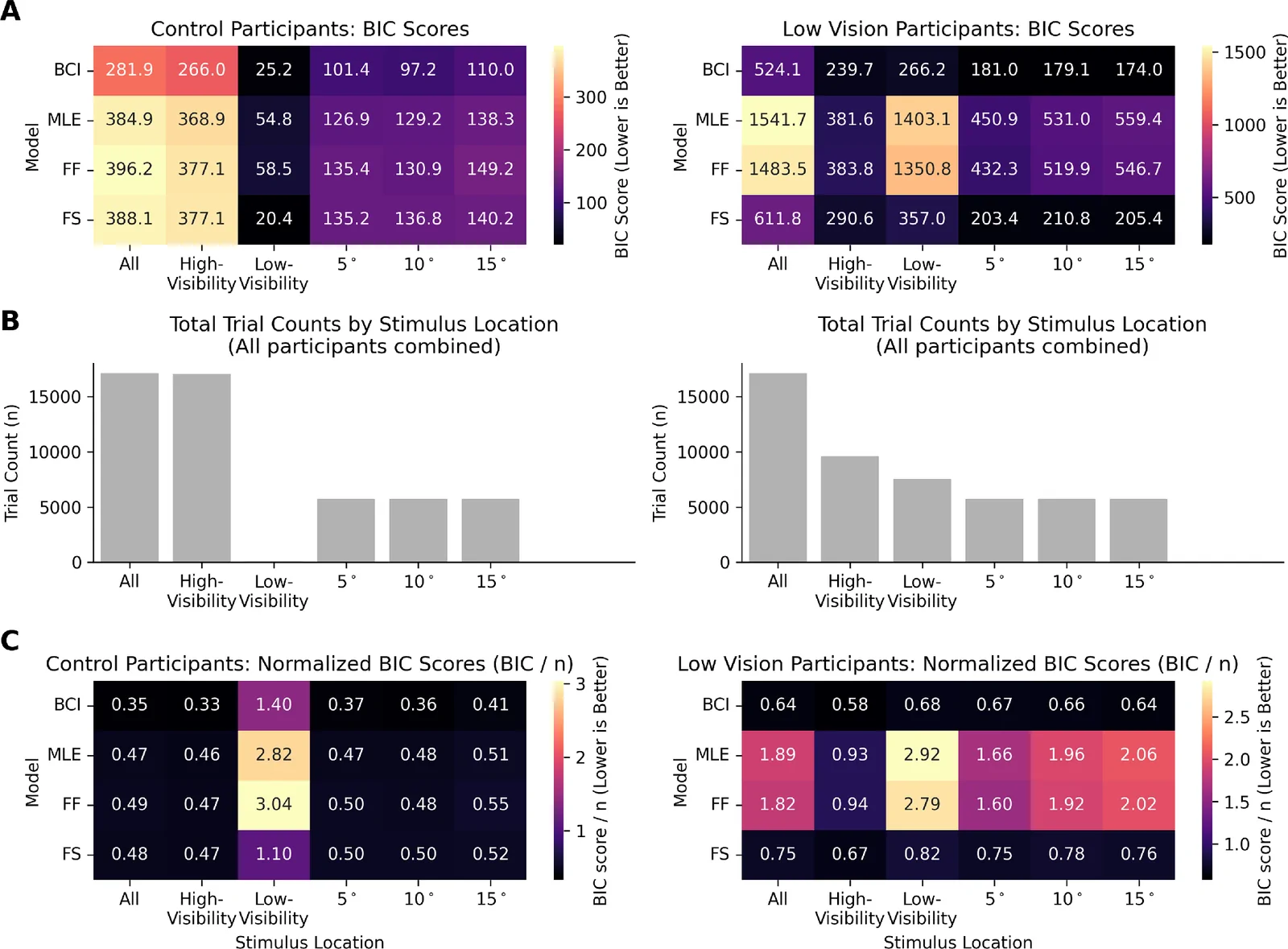
Model comparison and selection across stimulus location subsets. BIC evaluates model performance as a function of free parameters and predictive power; lower score means better model performance. **(A)** Raw BIC scores for the four candidate models (BCI, MLE, FF, and FS) across all spatial partitions for control (left) and low-vision (right) groups. Lower BIC scores indicate superior model performance, balancing goodness-of-fit with model parsimony. **(B)** Total trial counts (*n*) per stimulus location subset, illustrating the data volume used for each model fit. **(C)** Normalized BIC scores (*BIC*/*n*), accounting for the varying trial counts across subsets. This normalization reveals that while raw BIC scores may appear lower for low-visibility locations due to smaller sample sizes, the predictive power of the Bayesian framework is optimized at high-visibility locations. Note the consistent superiority of the BCI model (lowest *BIC*/*n*) across both groups in high-visibility conditions.

To determine which framework best characterized multisensory integration across the visual field, we compared the BIC scores of four candidate models across all stimulus location subsets (Figure 5A). In both groups, the BCI model consistently outperformed the MLE, forced-fusion (FF), and full-segregation (FS) models, yielding the lowest BIC scores across nearly all spatial partitions. However, model fits were systematically weaker for the low-vision group than for sighted controls (Figure 5A; BIC_*BCI*_: 524.15 ± 65.25 vs. 281.85 ± 35.99). The descriptive *R*^2^ values computed over response proportions were also high in both groups (controls: *R*^2^ = 1.00 ± 0.00; low vision: *R*^2^ = 0.96 ± 0.03), but these values should be interpreted cautiously because they are strongly influenced by control conditions (i.e., F0B0, F1B1) with highly predictable response distributions. Across both groups, the BCI framework maintained the superior balance of parsimony and goodness-of-fit.

As trial counts varied significantly across location subsets (Figure 5B), we evaluated model performance using normalized BIC scores (BIC/*n*; see Figure 5C). For both groups, the BCI model demonstrated peak performance at high-visibility locations (sighted control: 0.33 ± 0.05; low vision: 0.58 ± 0.12). Descriptive *R*^2^ values showed close agreement between observed and predicted response proportions in the sighted control group (*R*^2^ = 1.00 ± 0.00) and in the low-vision group (*R*^2^ = 0.97 ± 0.02), but these values do not constitute trial-level explained variance. In contrast, model fit was substantially reduced for low-visibility locations, with BIC/*n* scores increasing to 1.40 ± 0.21 for sighted controls and 0.68 ± 0.09 for low-vision participants. Correspondingly, response-proportion *R*^2^ values dropped to 0.73 ± 0.12 and 0.91 ± 0.04 for the sighted control and low-vision groups, respectively.

While model fits for sighted controls remained consistently robust (BIC/*n* ⩽ 0.55) across most spatial partitions, performance for low-vision participants degraded when spatial subsets contained heterogeneous visual reliability (e.g., BIC/*n* ⩾ 0.64 for the All, 5°, 10°, and 15° locations). Notably, two low-vision participants had no high-visibility locations and were excluded from subsequent subset analyses. To ensure the most reliable comparison of computational mechanisms, group-level analyses focused on high-visibility locations across the remaining 40 participants (21 sighted controls, 19 low vision), where model fits were robust in both groups. Because model performance at low-visibility locations was less ideal, particularly for the sighted control group, visibility-level comparisons were restricted to within-group analyses in the low-vision cohort.

#### Model fits and parameter estimation

**Figure 6.**
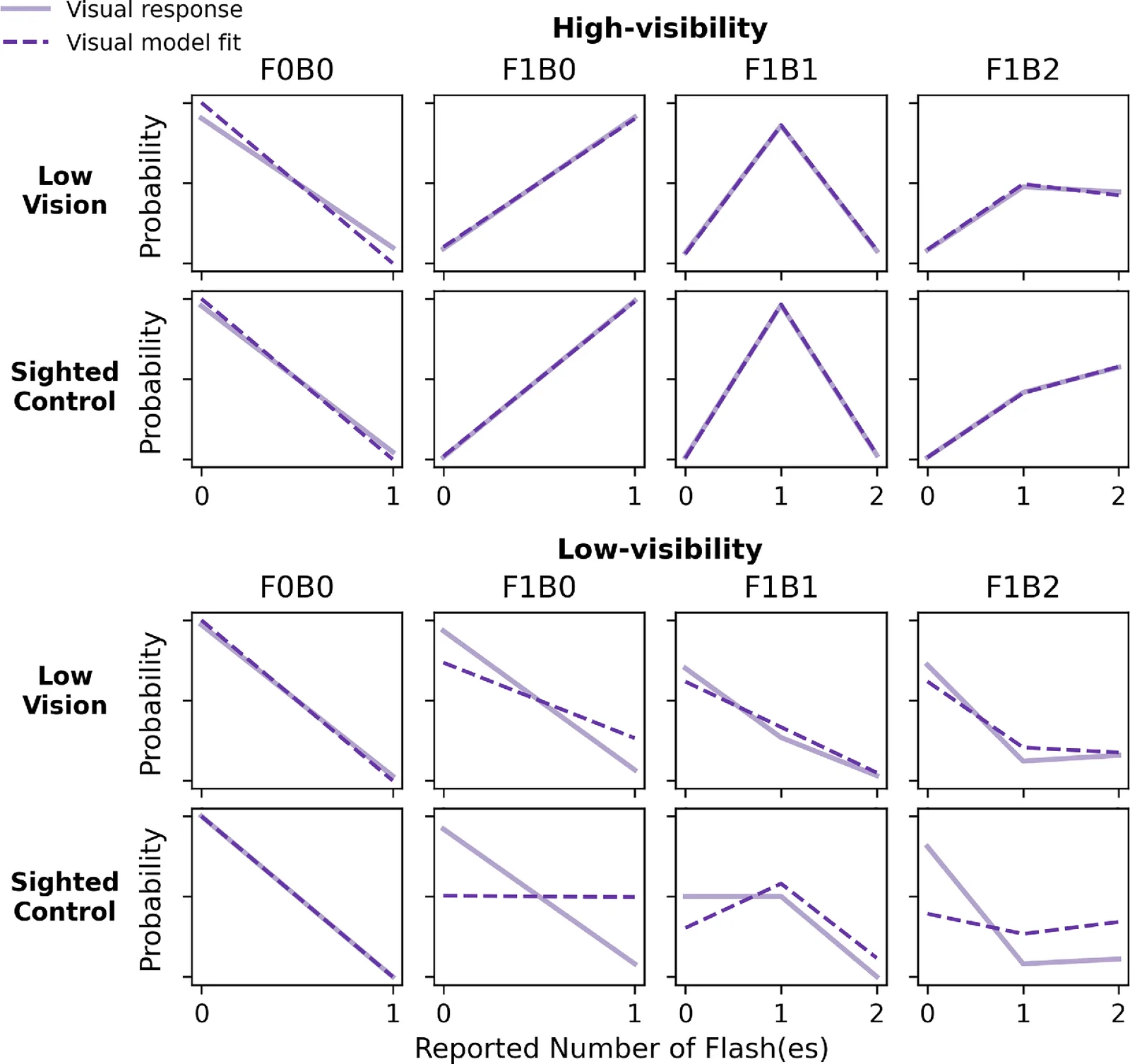
Group-level model fits and parameter distributions for high- and low-visibility locations. Mean response probabilities (solid lines) and BCI model predictions (dashed lines) for low-vision and sighted control groups across stimulus conditions, named using an FXBY convention (*X* = number of flashes, *Y* = number of beeps).

Both groups exhibited the characteristic SIFI in high-visibility locations, as evidenced by increased “2-flash” reports in the F1B2 condition (Figure 6). Qualitatively, sighted controls demonstrated a higher susceptibility to the illusion than the low-vision group, more frequently reporting two flashes when a single flash was accompanied by two beeps. Despite these behavioral differences, the BCI framework provided a robust fit for both groups, capturing the response distributions across all multisensory conditions, including the F1B2 illusion condition central to our analyses (Figure 6, top rows; Supplementary Figure S3).

Notably, despite the lower response-proportion *R*^2^ value (0.91 ± 0.04) at low-visibility locations in the low-vision group, the BCI model still captured the overall response pattern reasonably well (Figure 6, bottom rows). In contrast, poorer agreement was observed for sighted controls at low-visibility locations (*R*^2^ = 0.73 ± 0.12), likely reflecting insufficient sampling of low-visibility regions rather than a systematic change in inference structure (see Figure 5B). Because model fits for sighted controls in these regions were comparatively unstable, subsequent high- versus low-visibility analyses focused only on within-group comparisons in the low-vision cohort.

Comparison of internal parameter estimates (*p*_*common*_, σ_*V*_, σ_*P*_, and μ_*P*_) revealed limited evidence for group differences at high-visibility locations (Table 3). Although *p*_*common*_ was significantly lower in the low-vision group before correction (*p* = 0.01), the effect was modest after Bonferroni adjustment (*p*_*adj*_ = 0.04), with Bayesian analysis providing moderate evidence for a group difference (*BF*_10_ = 4.80). In contrast, sensory uncertainty (σ_*V*_) and prior mean (μ_*P*_) showed no evidence of group differences (*p*_*adj*_ = 1.00, *BF*_10_ ⩽ 0.40), providing support for parameter invariance across groups. Prior uncertainty (σ_*P*_) showed a nominal increase in the low-vision group (*p* = 0.04), but this effect did not survive correction (*p*_*adj*_ = 0.16), and the Bayes factor remained inconclusive (*BF*_10_ = 1.99). Overall, these findings suggest that under sufficiently visible conditions, the core sensory uncertainty structure of BCI remains largely preserved despite visual impairment, with only limited evidence for altered binding tendency.

**Table 3.** BCI parameter estimates and group comparisons across visibility levels.

Visibility	Parameter	Sighted control (Mean ± SE)	Low vision (Mean ± SE)	*t*	*p*_*uncorr*_	*p*_*adj*_	*BF*_10_
High	*p*_*common*_	0.71 ± 0.07	0.42 ± 0.08	−2.69	0.01	0.04	4.80
	μ_*P*_	1.38 ± 0.10	1.24 ± 0.19	−0.66	0.52	1.00	0.37
	σ_*P*_	0.74 ± 0.04	1.10 ± 0.16	2.20	0.04	0.16	1.99
	σ_*V*_	1.40 ± 0.21	1.64 ± 0.22	0.81	0.42	1.00	0.40
Low	*p*_*common*_	0.30 ± 0.13	0.20 ± 0.07	−0.66	0.53	1.00	0.50
	μ_*P*_	0.82 ± 0.44	0.30 ± 0.13	−1.11	0.32	1.00	0.65
	σ_*P*_	1.49 ± 0.62	0.91 ± 0.24	−0.87	0.42	1.00	0.56
	σ_*V*_	0.77 ± 0.07	1.08 ± 0.17	1.72	0.10	0.41	1.11

*Note*: High-visibility comparisons included *N*_*Control*_ = 21 and *N*_*LV*_ = 19. Low-visibility comparisons included *N*_*Control*_ = 5 and *N*_*LV*_ = 17. *p*_*adj*_ reflects Bonferroni correction for four comparisons within each visibility level. *BF*_10_ > 3 indicates evidence favoring a group difference; *BF*_10_ < 1 indicates evidence favoring the null hypothesis of group invariance.

Within the low-vision group, comparisons between high- and low-visibility locations revealed substantial changes in prior mean estimates (μ_*P*_), which were significantly higher at high-visibility locations (0.30 ± 0.13 vs. 1.24 ± 0.19, *t* = 4.19, *p*_*adj*_ < 0.001, *BF*_10_ = 61.68). In contrast, differences in binding tendency (*p*_*common*_) and sensory uncertainty (σ_*V*_) were only nominally significant before correction (*p* = 0.05 for both) and did not survive Bonferroni adjustment (*p*_*adj*_ = 0.18 and 0.21, respectively), with Bayes factors indicating inconclusive evidence (*BF*_10_ = 1.37 and 1.26). Prior uncertainty (σ_*P*_) did not differ between visibility levels (*p*_*adj*_ = 1.00, *BF*_10_ = 0.29), providing moderate evidence for invariance. Overall, these results suggest that the core causal inference structure remains relatively stable across visibility levels in low-vision participants, although reduced visibility may alter effective prior expectations and introduce noisier sensory estimates. However, these parameter differences should still be interpreted cautiously, as model fit quality decreased at low-visibility locations, indicating that the standard BCI assumptions may capture behavior less reliably under severely degraded visual conditions.

Finally, we evaluated the specific decision strategies employed by participants at high-visibility locations to map their internal causal estimates to a behavioral response (model averaging, probability matching, or model selection). Model averaging was the dominant strategy for both groups (sighted control: 52.4%; low vision: 47.4%), followed by probability matching (sighted control: 33.3%; low vision: 31.6%) and model selection (sighted control: 14.3%; low vision: 21.0%).

#### Model validation: Identifiability and recovery

To evaluate the reliability of our computational account, we performed a comprehensive validation of the BCI model's identifiability and parameter recovery.

First, we examined the identifiability of individual parameters. Under a high-visibility context, parameters were entirely independent, with no significant correlations observed between *p*_*common*_, σ_*V*_, μ_*P*_, and σ_*P*_ (see Supplementary Figure S4). This complete parameter identifiability confirms that the BCI framework successfully dissociates the observer's internal causal belief from their sensory noise.

Second, we conducted a parameter recovery analysis by simulating synthetic datasets based on our experimental design and participants’ best-fitting parameters. Initial recovery results indicated that all four parameters are well recovered (*p*_*common*_: *R*^2^ = 0.94, σ_*V*_: *R*^2^ = 0.79, σ_*P*_: *R*^2^ = 0.89, μ_*P*_: *R*^2^ = 0.90; see Supplementary Figure S5).

## Discussion

We investigated whether low vision alters susceptibility to the SIFI (Shams, Kamitani, & Shimojo, 2000) and whether illusion strength depends on local variations in visual sensitivity. Our results do not support a global “sensory compensation” account, which predicts that chronic visual degradation would lead to a compensatory increase in auditory reliance (Braun, 2016). Instead, our data suggest that multisensory integration is gated by local sensory reliability.

Low-vision participants were not more susceptible to the illusion than sighted controls; in fact, they were less likely to report the categorical “two-flash” percept. This suggests that the “double-flash” illusion is not a default response to visual uncertainty but rather a sophisticated interaction that requires a minimum threshold of visual signal to manifest. When visual input is too degraded, the brain may fail to form a sufficiently strong visual representation for the auditory signal to “split,” leading to the reduced susceptibility we observed. Indeed, a similar study investigating factors affecting the susceptibility of SIFI showed that SIFI was robust regardless of the spatial proximity of the audiovisual stimuli but depended on local visual sensitivity (Kumpik, Roberts, King, & Bizley, 2014).

A key finding of this study is that illusion strength was more robustly predicted by local flash-detection accuracy than by spatial eccentricity. While eccentricity-dependent trends approached marginal significance (*p* < 0.10), they were overshadowed by the direct relationship between a participant’s ability to detect a single flash and their susceptibility to the illusory second flash. This indicates that the “Bayesian brain” does not integrate information based on a fixed spatial map but rather dynamically weighs cues based on the immediate reliability of the retinal signal at that specific coordinate.

Interestingly, the lack of a robust eccentricity trend was observed not only in the low-vision group but also among sighted controls. While classic models of multisensory integration predict increased auditory reliance as visual stimuli move into the periphery (Chen, Maurer, Lewis, Spence, & Shore, 2017; Chang et al., 2023), our results align more closely with recent findings suggesting that eccentricity alone does not dictate illusion susceptibility (Gavin, Hirst, & McGovern, 2022). We propose that previously reported eccentricity-dependent effects may serve as a proxy for sensory reliability. The absence of a spatial gradient in our healthy cohort—who exhibited near-ceiling flash-detection accuracy across all tested locations—further reinforces our primary conclusion: The SIFI is gated by sensory reliability rather than spatial position. This is supported by a recent computational simulation demonstrating that even when the prior integration tendency (*p*_*common*_) remains constant, the frequency of the illusion increases as the reliability of vision (σ_*V*_) decreases (Zhu, Beierholm, & Shams, 2024b). Conversely, in the low-vision group, the reduced susceptibility to the categorical “double-flash” percept suggests that when visual uncertainty is too high, the brain may fail to form a sufficiently stable visual representation for the auditory signal to influence (Rahnev & Denison, 2018). Consequently, the differences in illusion susceptibility observed in low vision are not an architectural change in integration but a functional consequence of how the brain manages extreme visual ambiguity.

The exploratory BCI modeling provides the most nuanced insight into this process. When fitted to trials at high-visibility locations, the model produced strong fits for both the low-vision and sighted control groups, suggesting that multisensory cue combination and segregation remain broadly consistent with Bayesian inference when visual signals are sufficiently reliable. Interestingly, low-vision participants showed a lower *p*_*common*_ relative to sighted controls, indicating a weaker prior tendency to infer a common cause across sensory modalities. One possible explanation is that reduced multisensory exposure in daily life due to chronic visual impairment may weaken the development or maintenance of multisensory binding priors (Senna et al., 2021; Scheller, Proulx, de Haan, Dahlmann-Noor, & Petrini, 2021). However, this effect should be interpreted cautiously, as only *pcommon* survived multiple-comparison correction and the broader parameter pattern remained largely stable across groups.

When comparing fitted BCI parameters between high- and low-visibility locations within the low-vision group, we observed a significantly lower μ_*P*_ at low-visibility locations, suggesting that participants expected fewer visual events when visual input became unreliable. In contrast, we did not observe statistically reliable changes in σ_*V*_, the parameter theoretically associated with visual sensory uncertainty, or strong evidence for changes in binding tendency (*p*_*common*_). Importantly, these parameter estimates should be interpreted with caution. The current experiment was optimized for spatial sampling across many visual-field locations rather than exhaustive model fitting and therefore did not include the full factorial stimulus space of multiple flashes and beeps (e.g., 0, 1, and 2 flashes/beeps). Furthermore, model fit quality decreased at low-visibility locations, suggesting that behavior under severe visual degradation may be noisy or not Bayes-optimal. In these regimes, uncertainty may become underconstrained or partially absorbed into prior-related parameters. Nevertheless, the absence of strong changes in *p*_*common*_ across visibility levels suggests that the fundamental causal inference structure remains relatively stable despite degraded vision.

Finally, our behavioral findings refine, rather than contradict, neural evidence showing cross-modal recruitment of occipital cortex in visual impairment (Cunningham et al., 2015; Collignon, Voss, Lassonde, & Lepore, 2009; Kupers & Ptito, 2014). While auditory signals may indeed recruit occipital cortex when some visual function remains, our results demonstrate that such reorganization does not manifest as a global amplification of multisensory interactions. Instead, auditory influence on perceived numerosity is strictly gated by the reliability of local visual input.

Together, these results show that chronic sensory degradation does not automatically lead to compensatory multisensory enhancement. Rather, sound-induced increases in perceived flash count emerge only when visual input is sufficiently reliable to support a stable representation; as vision fails, the brain’s alignment with Bayesian-optimal predictions becomes systematically weaker and more variable. Ultimately, the SIFI is a reliability-dependent interaction, and group-level computational comparisons must be interpreted through the lens of local visual accuracy and the resulting limits of model adequacy.

While the present study provides robust evidence for shared multisensory integration strategies between sighted and low-vision populations, several limitations warrant consideration.

First, although we observed significant main effects of auditory condition and visual visibility, several interactions involving spatial eccentricity reached only marginal significance (*p* < 0.10). Specifically, the negative coefficients for the *Condition* × *Eccentricity* interactions suggest a trend where visual accuracy degrades more sharply in the periphery. As a formal a priori power analysis was not performed for these specific higher-order interactions, and no explicit stopping rule was defined for data collection, it is possible that our sample size (*N* = 42) was underpowered to robustly detect these subtle spatial effects. Future studies with larger cohorts or targeted high-eccentricity paradigms may be necessary to determine if these trends cross the threshold of statistical significance.

Second, our computational approach utilized a BCI model with certain fixed parameters, such as the auditory noise (σ_*A*_). While this allowed for a more parsimonious model that successfully captured group-level trends, individual differences in sensory processing might be better captured by allowing these parameters to vary. It should be noted that the higher BIC scores observed in the low-visibility locations are partially attributable to the mathematical properties of the likelihood function; as internal noise increases, the maximum achievable likelihood naturally decreases. Future iterations of multisensory models might explore non-Gaussian likelihoods or incorporate additional parameters to account for the specific stochastic nature of clinical vision loss.

Third, while the current study focuses on temporal multisensory integration via SIFI, future research could employ complementary spatial illusions, such as the Ventriloquist effect (Alais & Burr, 2004; Richards, Goltz, & Wong, 2018; Orchard-Mills, Van der Burg, & Alais, 2016). Given that the Ventriloquist illusion typically relies more heavily on visual spatial precision, investigating this effect in low-vision populations would clarify whether the observed preservation of Bayesian integration logic extends from the temporal to the spatial domain.

Furthermore, our low-vision cohort was heterogeneous in diagnosis, the spatial extent of field loss, and disease duration, likely contributing to the observed variability in both behavior and model fits. While our results suggest a commonality in integration strategies, the specific nature of a visual deficit (e.g., central vs. peripheral loss) may exert unique pressures on the multisensory system that categorical grouping might obscure.

Finally, although eye tracking would have allowed direct verification of fixation stability and retinal locus of stimulation, standard calibration is often infeasible for low-vision individuals. At the time of data collection, our laboratory did not yet have accessible calibration tools (e.g., skipped-calibration modes, nonfoveal target methods). We therefore relied on task structure and instructions rather than direct gaze measurement. Future work incorporating low-vision-adaptive eye tracking will be crucial for linking local retinal function, fixation behavior, and multisensory integration.

## Conclusions

This study examined how chronic, pathological visual impairment shapes multisensory inference in the sound-induced flash illusion. Contrary to a global “sensory compensation” account, low vision did not lead to a systemic increase in illusion susceptibility. Instead, our results demonstrate that audiovisual interactions are strictly gated by local sensory reliability. Perceived flash numerosity was more robustly predicted by a participant’s ability to detect a single flash than by their clinical group membership or spatial eccentricity. This suggests that the “double-flash” percept is not a default response to visual noise but a sophisticated interaction that requires a minimum threshold of visual signal to manifest.

Our exploratory BCI modeling provided a nuanced “stress test” of the brain’s computational architecture. While global fits were weaker and more variable in the low-vision cohort, restricting the analysis to high-visibility locations yielded the strongest BIC performance and close agreement between observed and predicted response proportions. We interpret the high response-proportion *R*^2^ values descriptively rather than as evidence that the model explained trial-level behavioral variance, because the metric is influenced by highly predictable control conditions. Even with this caveat, the visual match between observed and predicted response distributions, especially for the F1B2 illusion condition, supports the conclusion that behavior at high-visibility locations remains broadly consistent with BCI. The poor model fits at low-visibility locations likely reflect a condition of extreme ambiguity, where the brain may rely more heavily on non-Bayesian heuristics or stochastic guessing.

Together, these results demonstrate that degraded vision does not automatically amplify multisensory integration. Instead, Bayesian-like patterns of multisensory inference appear most stable when observers have a sufficient threshold of usable sensory input. These findings suggest that potential neural reorganization in low vision does not necessarily overwrite the core computational principles of the nervous system. Future work combining low-vision-adaptive eye tracking with models that account for trial-level variability will be essential for further mapping the transition from optimal integration to heuristic-based perception under chronic sensory uncertainty.

## Supplementary Material

Supplement 1
