# Supplementary material for: Did you see the sound? A Bayesian assessment on cross-modal perception in low vision
Source: J Vis. 2026 Aug 25;26(8):7. doi: 10.1167/jov.26.8.7 (PMC13543619; doi:10.1167/jov.26.8.7)
Supplement: Supplementary file 1 [file jovi-26-8-7_s001.pdf]

Supplementary Information: Did you see the sound? A Bayesian Assessment on Crossmodal Perception in Low Vision

Ailene Y. C. Chan, Noelle R. B. Stiles, Carmel A. Levitan, Armand R. Tanguay, Jr., Shinsuke Shimojo

Appendix S1: Beep Detection Task and Auditory Parameter Validation

This task was performed by a subset of 16 newly recruited age-matched controls following the completion of data collection in the low-vision cohort. The primary objective was to empirically validate the auditory likelihood parameter (σ_A) used in our BCI modeling.

Methods

The visual and auditory stimuli in this task were identical to that in the main experiment (Visual Flash Detection Task, Illusory Double Flash Task). Visual stimuli were (circular white discs (2° in diameter) presented on a black background at 100% contrast for one frame (16.7 ms at 60 Hz). Auditory stimuli were pure tones (800 Hz, 7 ms duration). Six audiovisual combinations of flashes (0 or 1) and beeps (0, 1, or 2) were tested. Stimulus timing followed the same parameters as described in the main text (see Fig. 1B–E).

Parameter Estimation

To derive σ_A, we fit a unisensory Gaussian observer model to the numerosity responses. To ensure the estimation was not biased by visual parameters, we utilized a "flat-visual" BCI framework where:

- $\sigma_V =$ 4000: Fixed to ensure the visual likelihood provided no numerosity information.
- $p_{common} = 0$: Fixed to assume independent causes, effectively bypassing the fusion logic of the BCI model.
- $\mu_P = 0$, $\sigma_P =$ 4000: Fixed to ensure a flat, uninformative prior
- σ_A: Remained the only free parameter.

Results

This empirical result confirms that the auditory sensitivity ($\sigma_A = 0.19 \pm 0.08$) of our age-matched control participants is highly consistent with the $\sigma_A = 0.2$ value established in previous studies using identical stimuli (Chan et al., 2025). This high precision is further supported by the near-ceiling performance observed in our beep-detection task. Given this cross-study consistency and the minimal variance observed in the control subset, we fixed σ_A at 0.2 across both experimental groups. This constraint reduces the model's complexity and facilitates a direct, parsimonious

comparison of visual reliability σ_V and causal priors p_{common} between the Sighted Control and Low Vision populations.

Mean Accuracy of Perceiving Beeps (N=16)

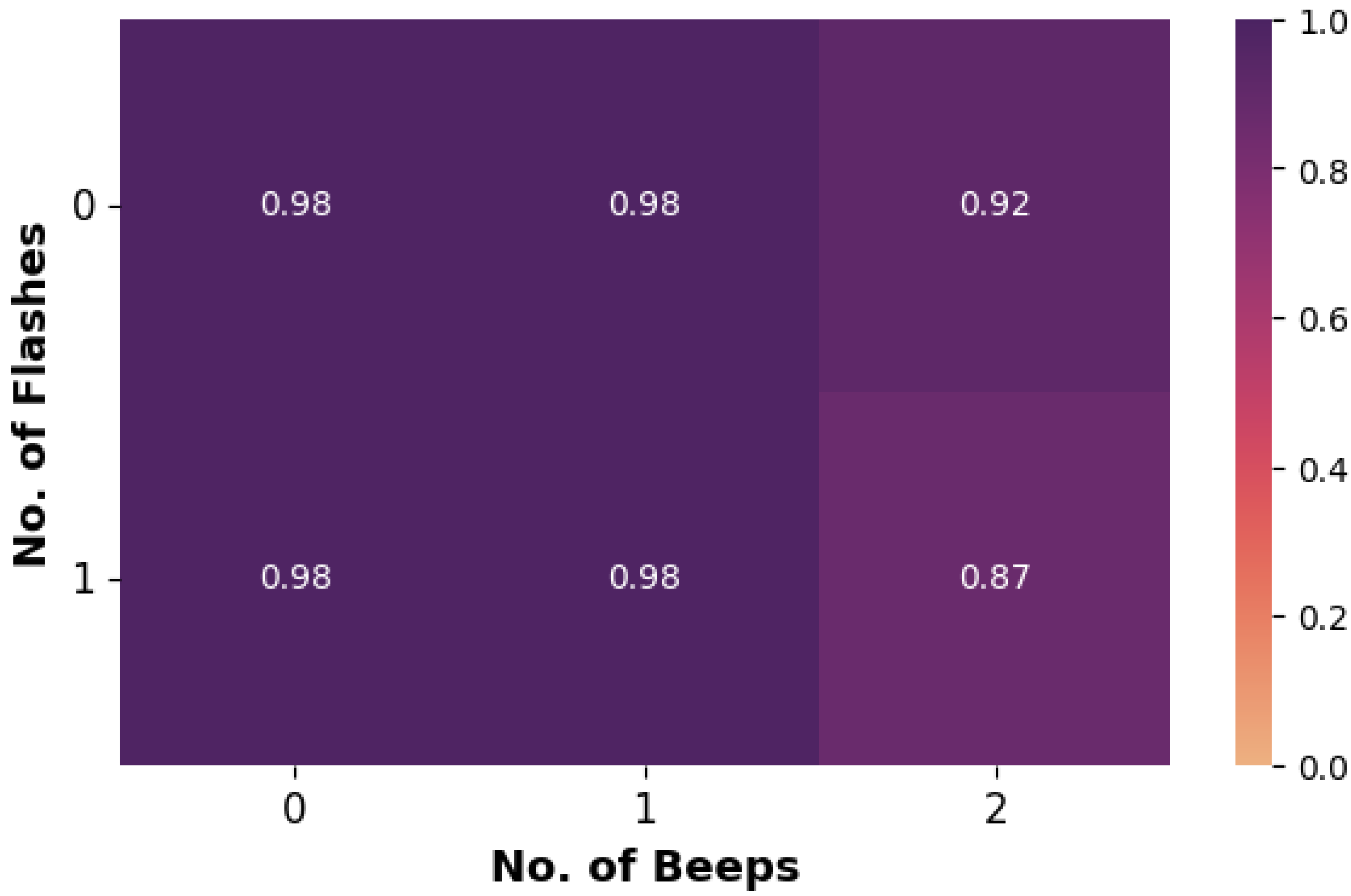

Heatmaps show accuracy in reporting the true number of beeps (0/1/2) across conditions. Performance is near ceiling (1.0) across conditions, suggesting that number of flashes present has little influence on beep detection accuracy.

References

Chan, A. Y. C., Stiles, N. R. B., Levitan, C. A., Wu, D.-A., Tanguay, A. R., & Shimojo, S. (2025). Bayesian Causal Inference Accounts for Multisensory Filling-In at the Blind Spot. *bioRxiv*, 2024.11.15.623713. https://doi.org/10.1101/2024.11.15.623713

Figure S1. Sensitivity Analysis for different σA values

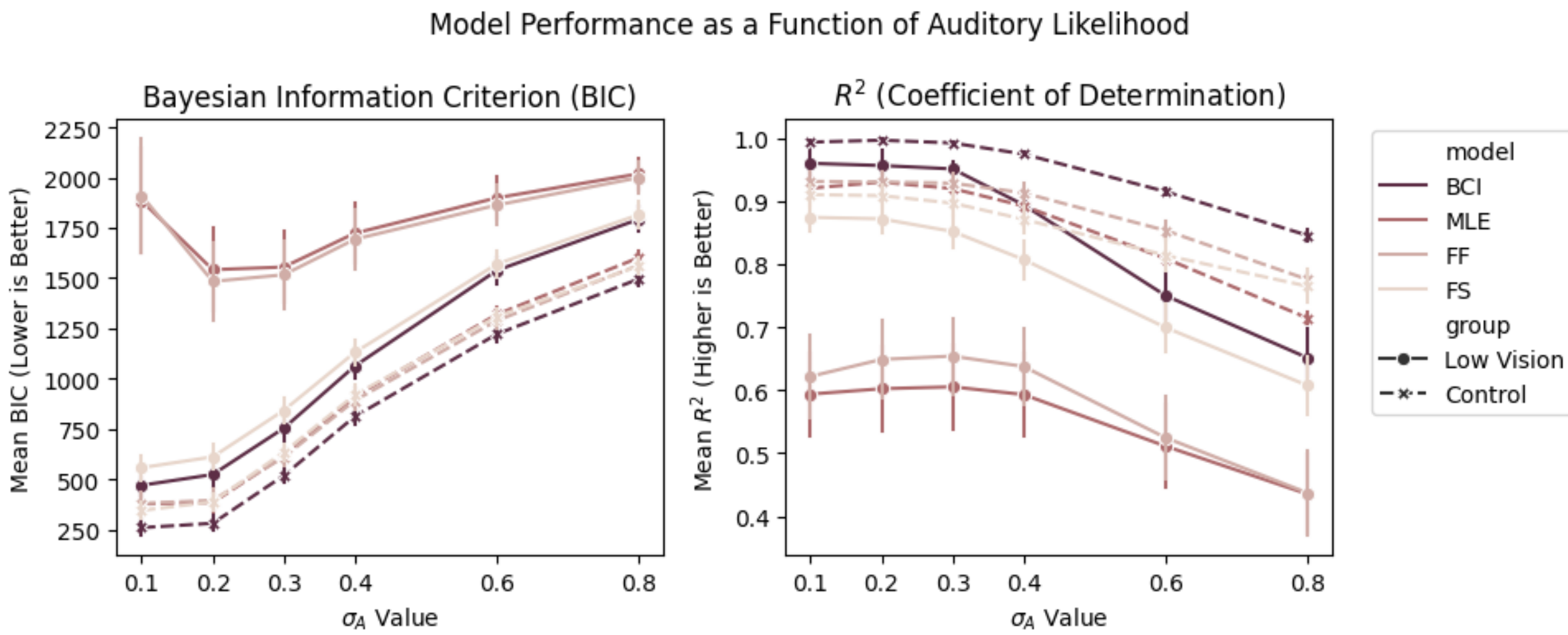

To validate the selection of the fixed auditory likelihood parameter **(**σA), we performed a parameter sweep across a range of values (0.1, 0.2, 0.3, 0.4, 0.6, 0.8). By visual inspection, it was apparent that 0.1-0.3 yield best model performance (low BIC scores and high R2). We performed Wilcoxon sign-ranked tests to compare BIC and R2 between 0.1 and 0.2, and 0.2 and 0.3. After correcting for multiple comparisons (Bonferroni), Model_3 performs significantly worse than Model_2, while Model_1 has significantly lower BIC scores than Model_2, there is no meaningful difference in R2.

Figure S2. Trial-level response distributions for Sighted Control and Low Vision groups.

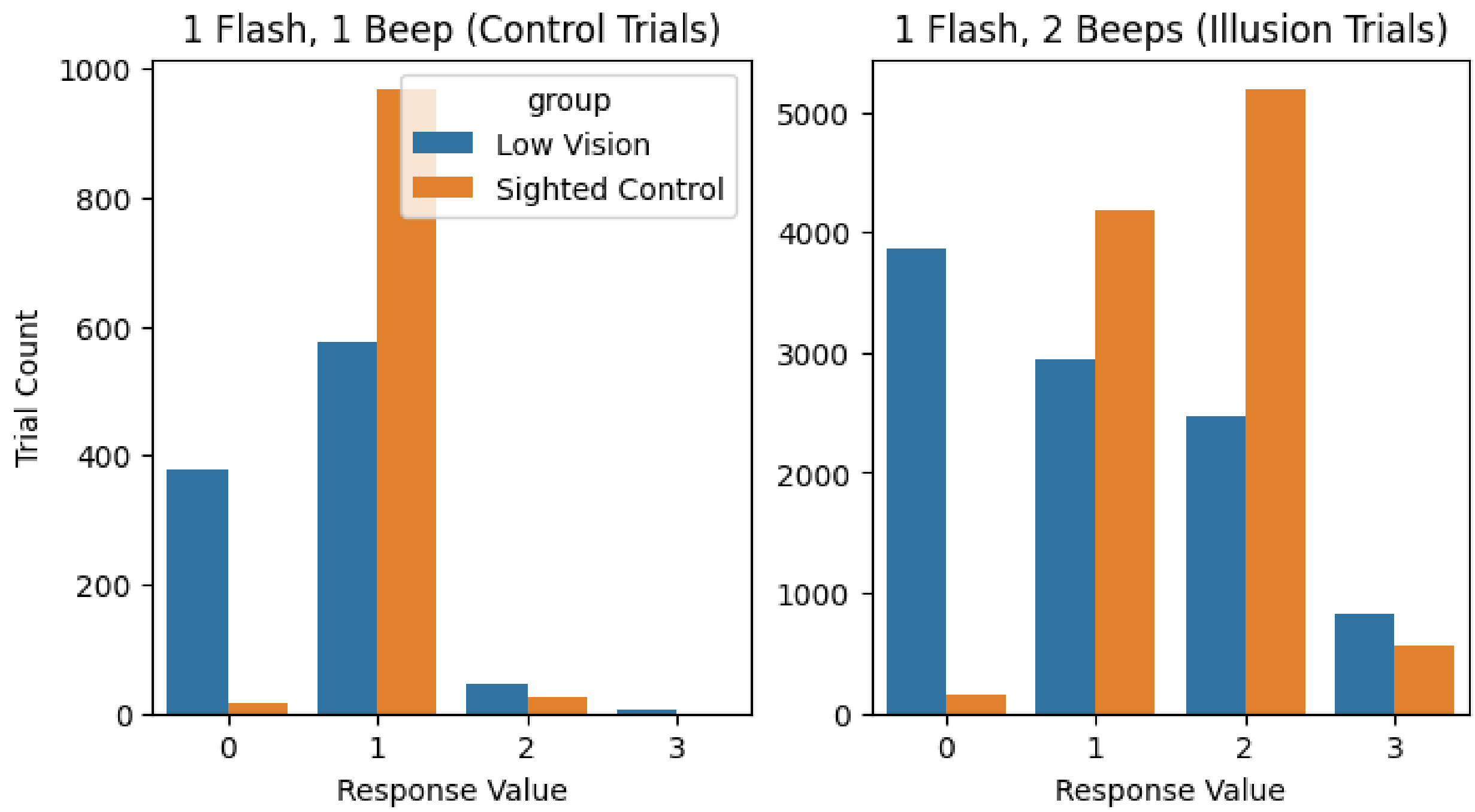

Bars represent the total count of trials for each response value (number of flashes perceived).

Left: 1 Flash, 1 Beep (Control Trials) – While Sighted Controls show high detection consistency (peaking at 1), the Low Vision group exhibits a substantial number of “misses” (0 flashes), indicating a lower sensory detection threshold.

Right: 1 Flash, 2 Beeps (Illusion Trials) – Inaccuracy in Sighted Controls is driven by a shift toward reporting 2 flashes (the sound-induced flash illusion). In the Low Vision group, inaccuracy is split between “misses” (0) and “illusions” (2). This bimodal error distribution explains why the Low Vision group's mean perceived flashes can remain near 1.0 despite significantly lower accuracy than controls.

Figure S3. Group average Bayesian Causal Inference model fits for all locations.

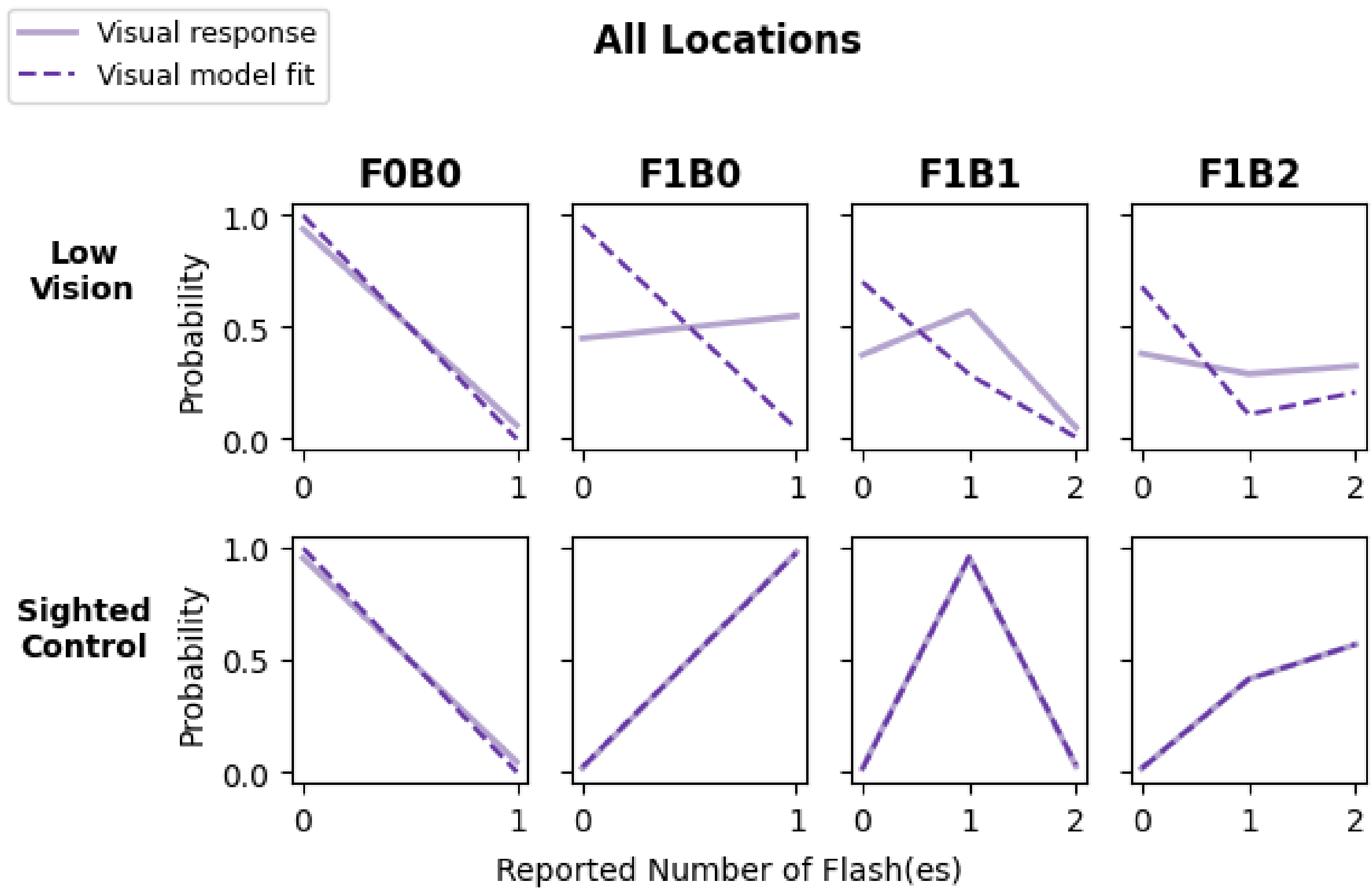

Visual response distributions (solid lines) and corresponding model-predicted distributions (dashed lines) are shown for four conditions (F0B0, F1B0, F1B1, F1B2). The top row depicts group average for low vision participants, and the bottom row depicts group average for sighted controls. Each panel shows the probability of reporting 0–2 flashes for that condition.

Figure S4. Parameter Identifiability Matrix.

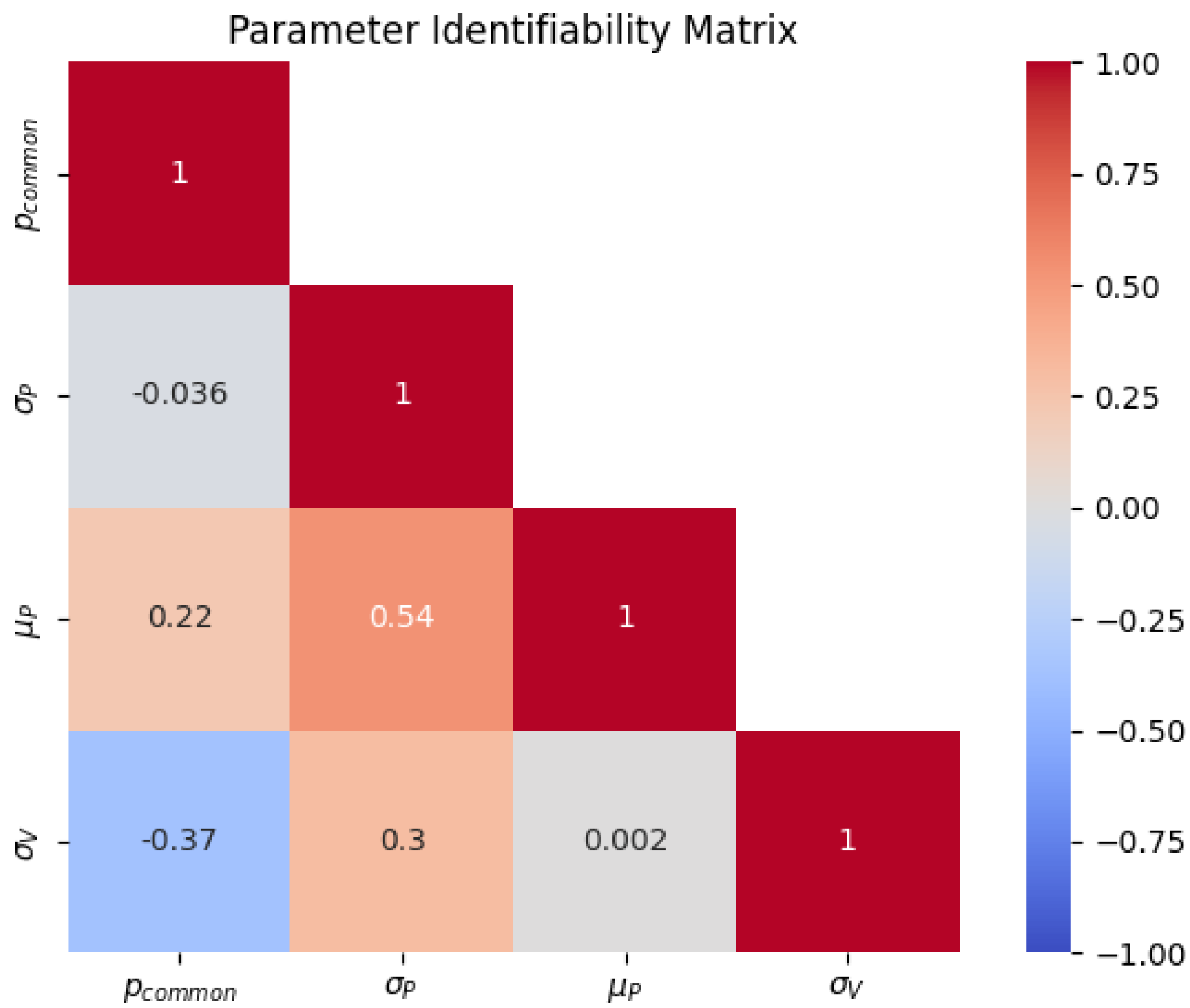

Correlation matrix for BCI parameters in high-visibility locations. Most parameter pairs showed weak correlations ($|r| < 0.4$), including the relationship between p_{common} and σ_V ($r = -0.37$), suggesting that visual uncertainty and binding tendency were largely dissociable within the fitting procedure. A moderate correlation was observed between σ_P and μ_P ($r = 0.54$), indicating partial dependence between prior width and prior mean estimates. Overall, the absence of strong inter-parameter correlations supports reasonable parameter identifiability within the BCI framework, particularly for the key distinction between sensory precision and causal binding tendency

Figure S5. Parameter recovery analysis.

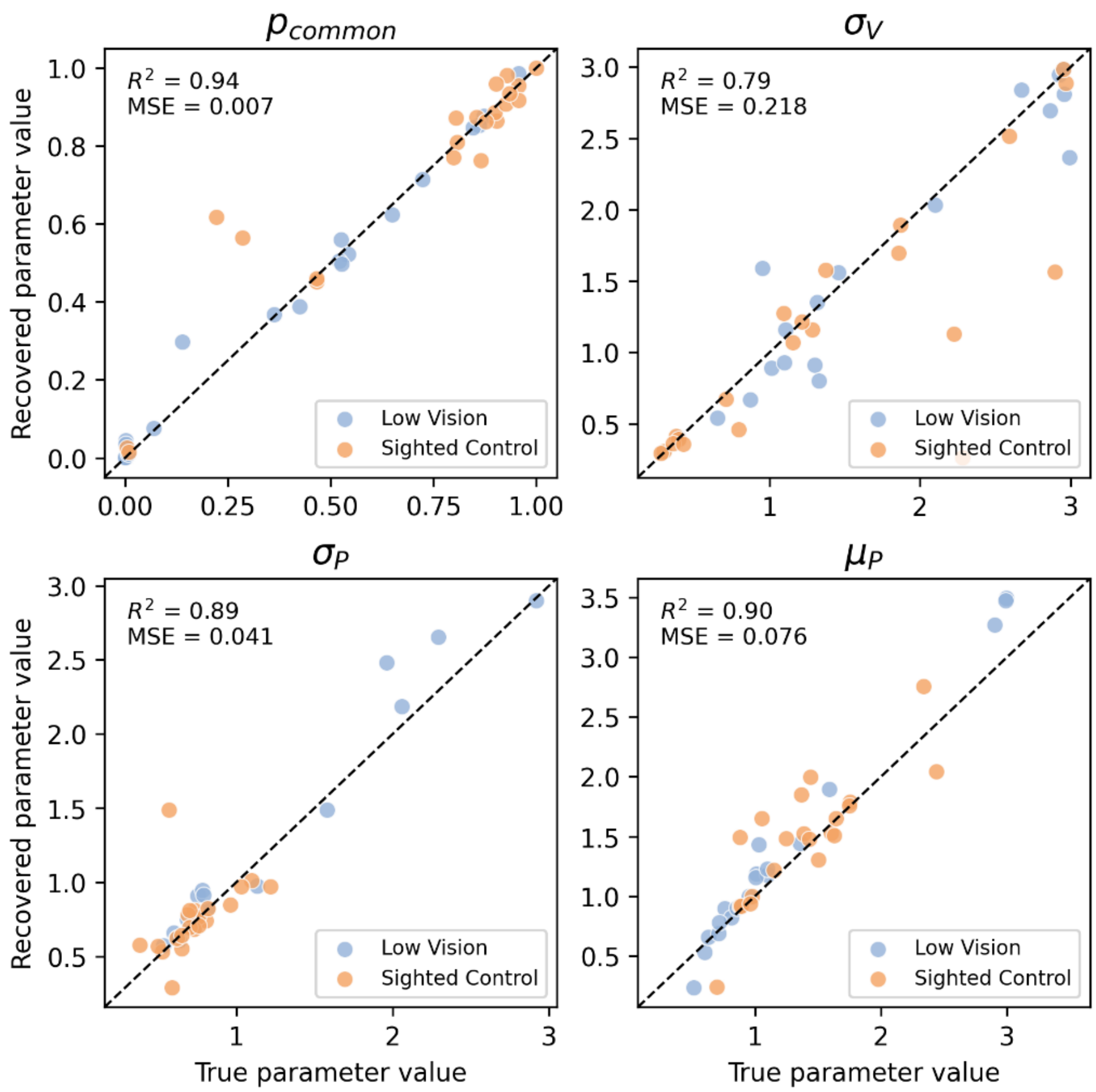

Correlation between ground-truth and recovered parameter values for the BCI model fitted at high-visibility locations. Synthetic datasets were generated using each participant's estimated parameters and subsequently re-fitted to evaluate the model's identifiability and the study design's robustness. In each panel, the recovered values are plotted against the ground truth (black dashed lines indicate the identity $y = x$). High R^2 and low Mean Squared Error (MSE) were observed for all parameters, indicating these parameters were reliably recovered.
